# A multiscale modelling approach to understand atherosclerosis formation: A patient-specific case study in the aortic bifurcation

**DOI:** 10.1177/0954411917697356

**Published:** 2017-04-21

**Authors:** Mona Alimohammadi, Cesar Pichardo-Almarza, Obiekezie Agu, Vanessa Díaz-Zuccarini

**Affiliations:** 1 Department of Mechanical Engineering, University College London, London, UK; 2 Vascular Unit, University College London Hospitals, London, UK

**Keywords:** Mathematical modelling, multiscale, atherosclerosis, patient-specific, calcification

## Abstract

Atherogenesis, the formation of plaques in the wall of blood vessels, starts as a result of lipid accumulation (low-density lipoprotein cholesterol) in the vessel wall. Such accumulation is related to the site of endothelial mechanotransduction, the endothelial response to mechanical stimuli and haemodynamics, which determines biochemical processes regulating the vessel wall permeability. This interaction between biomechanical and biochemical phenomena is complex, spanning different biological scales and is patient-specific, requiring tools able to capture such mathematical and biological complexity in a unified framework. Mathematical models offer an elegant and efficient way of doing this, by taking into account multifactorial and multiscale processes and mechanisms, in order to capture the fundamentals of plaque formation in individual patients. In this study, a mathematical model to understand plaque and calcification locations is presented: this model provides a strong interpretability and physical meaning through a multiscale, complex index or metric (the penetration site of low-density lipoprotein cholesterol, expressed as volumetric flux). Computed tomography scans of the aortic bifurcation and iliac arteries are analysed and compared with the results of the multifactorial model. The results indicate that the model shows potential to predict the majority of the plaque locations, also not predicting regions where plaques are absent. The promising results from this case study provide a proof of concept that can be applied to a larger patient population.

## Introduction

Atherosclerosis in any of its clinical manifestations (e.g. heart attack, stroke and peripheral arterial disease), commonly known as ‘hardening or furring of the arteries’, is the leading cause of morbidity and mortality worldwide. A chronic condition, the disease originates from the accumulation of lipids in the arterial wall, triggering an inflammatory response and subsequent evolution and transformation into fibrous tissue.

Atherosclerotic-prone sites are mainly located in the intima of many middle-sized and large arteries, particularly where the vessels divide. Most likely this is influenced by the nature of the blood flow, since areas exposed to normal shear stress appear to be protected.^1^ Lipid accumulation and fibrosis is a complex, multifactorial process that occurs as a result of a number of factors at several spatial and temporal scales. The bewildering complexity of the disease is influenced by genetic, metabolic, immune, inflammatory, haemodynamic, anatomical and environmental variables affecting its progression, among others.

Additionally, calcification seems to form specifically in the muscle cells adjacent to atheromas and on the surface of atheroma plaques and tissue.^2^ In time, this leads to extracellular calcium deposits between the muscular wall and outer portion of the atheromatous plaques. With the atheromatous plaque interfering with the regulation of the calcium deposition, it accumulates and crystallises.

The initiation and progression of atherosclerosis has been linked with the infiltration and accumulation of macromolecules, for example, low-density lipoproteins (LDLs), in the arterial wall. Once LDL in circulation manages to enter the wall and is oxidised by reactive oxygen species (ROS), the main inflammatory processes related to the disease are triggered and the formation of plaque starts. The innermost layer of the arterial wall, the endothelium (a thin layer of endothelial cells that form the interface between circulating blood and the rest of the arterial wall), plays a very relevant role in the arterial mass transport.

Transendothelial mass transport can occur through paracellular interendothelial junctions and/or transcellular aqueous channels.^3^ More specifically, there are three potential pathways for transport of macromolecules across the endothelium:^4^ transcytosis in vesicles, paracellular transport through the breaks in the tight junction strand and a ‘leaky junction’ pathway associated with cells undergoing mitosis or apoptosis.^5^ It has been proposed that these leaky junctions are the primary pathway for LDL transport.^6^ In fact, results from in vitro studies,^7^ using bovine aortic endothelial cells (BAECs), showed that leaky junctions are the dominant transport pathway for LDL under convective conditions, accounting for more than 90% of the LDL transport, whereas the vesicular pathway accounted for the remainder (∼9%).

Some haemodynamic factors have been linked to the initiation and progression of atherosclerosis, which also affect endothelial cell apoptosis.^5^ For example, Frangos et al.^8^ showed how regions of disturbed flow and low shear stress increase the risk of atherosclerosis progression. Other studies^9–11^ showed how irregular flow or increase in shear stress increases or reduces endothelial cell apoptosis, respectively.

As previously described, atherogenesis is the result of an incredible network of interlinked and complex processes: ever-evolving, subject-specific and occurring at multiple levels from molecules to the organism level; these processes are not separate, but parts of a gigantic multiphysics and multiscale system. The fact that we are still grappling with the understanding of the disease has an enormous cost for patients and presents a tremendous challenge for clinicians who, nevertheless, must deal with the disease (and its consequences) every single day. This is a case where mathematical modelling can offer much needed help. Mathematical modelling provides an efficient and elegant way to combine observations and the physical description of specific mechanisms, and it is therefore a very powerful instrument for the design of new in silico tools to integrate quantitative information with our current understanding of biology and physiology.

It is logical that from an engineering point of view, much work in atherosclerosis modelling has been focused on the role of haemodynamic variables in plaque formation. Although this research is mathematically elegant and has produced some seminal findings in the area,^12^ it has been traditionally disconnected from cellular and molecular research. By breaking down the complex processes involved in the disease into manageable and disjointed parts, we are automatically constraining these mathematical models in ways we do not fully comprehend, with research results offering a partial or obscure view that might even be misleading. Given the incredible complexity of the atherogenesis process, these haemodynamic analyses, on their own, have been inconclusive^13^ and a clear metric for plaque location remains elusive.

Based on the multifactorial and multiscale nature of the disease as described above and the strong literature findings^14–16^ supporting a key role of the endothelium in the atherogenesis process, the aim of this article is to use a multiscale, mechanistic model of endothelial mechanotransduction to explain calcification and atherosclerotic plaque areas in a patient-specific case and to use a multiscale, multifactorial and interpretable metric for plaque location. This model will include key haemodynamic variables affecting endothelial behaviour (in particular wall shear stress (WSS) and oscillatory shear index (OSI)) and a multiscale model of the endothelium and the penetration mechanisms of LDL into the arterial wall. This article is organised as follows: section ‘Methods’ presents the methods and simulation details, along with the reconstructed computed tomography (CT) scan data showing the regions of calcification/plaque formation. Simulation results of the model will be presented in section ‘Results’ and compared to atherosclerotic plaques and calcifications indicated in the original CT scans of a patient-specific aortic bifurcation. The discussion and conclusion of this work will be presented in sections ‘Discussion’ and ‘Conclusion’, respectively.

## Methods

### Image processing

CT scans of the aortic bifurcation of a 54-year-old patient were used in this study (ethics 13/EM/0143). This patient was originally scanned due to an aortic dissection (AD). The dissection was located in the descending aorta and persisted until approximately 10 cm proximal to the aortic bifurcation (the focus of this article), wherein the false and true lumina reconnected to form a single lumen. Approximately 100 CT images were imported into ScanIP image processing software (Simpleware Ltd, UK). These images provided a stack of two-dimensional (2D) planes with a resolution of 0.7 mm/pixel and 0.7 mm between planes.

For the sake of efficiency, the entire stack was cropped to only include the region of interest (the aortic bifurcation), and all external regions, such as the CT scan bed and soft tissue, were removed to increase the image processing efficiency. The imported images were converted to 8 bit and therefore the intensities ranged between 0 and 255. The segmentation was performed for the lumen and both calcification and plaque locations.

**Figure 1.**
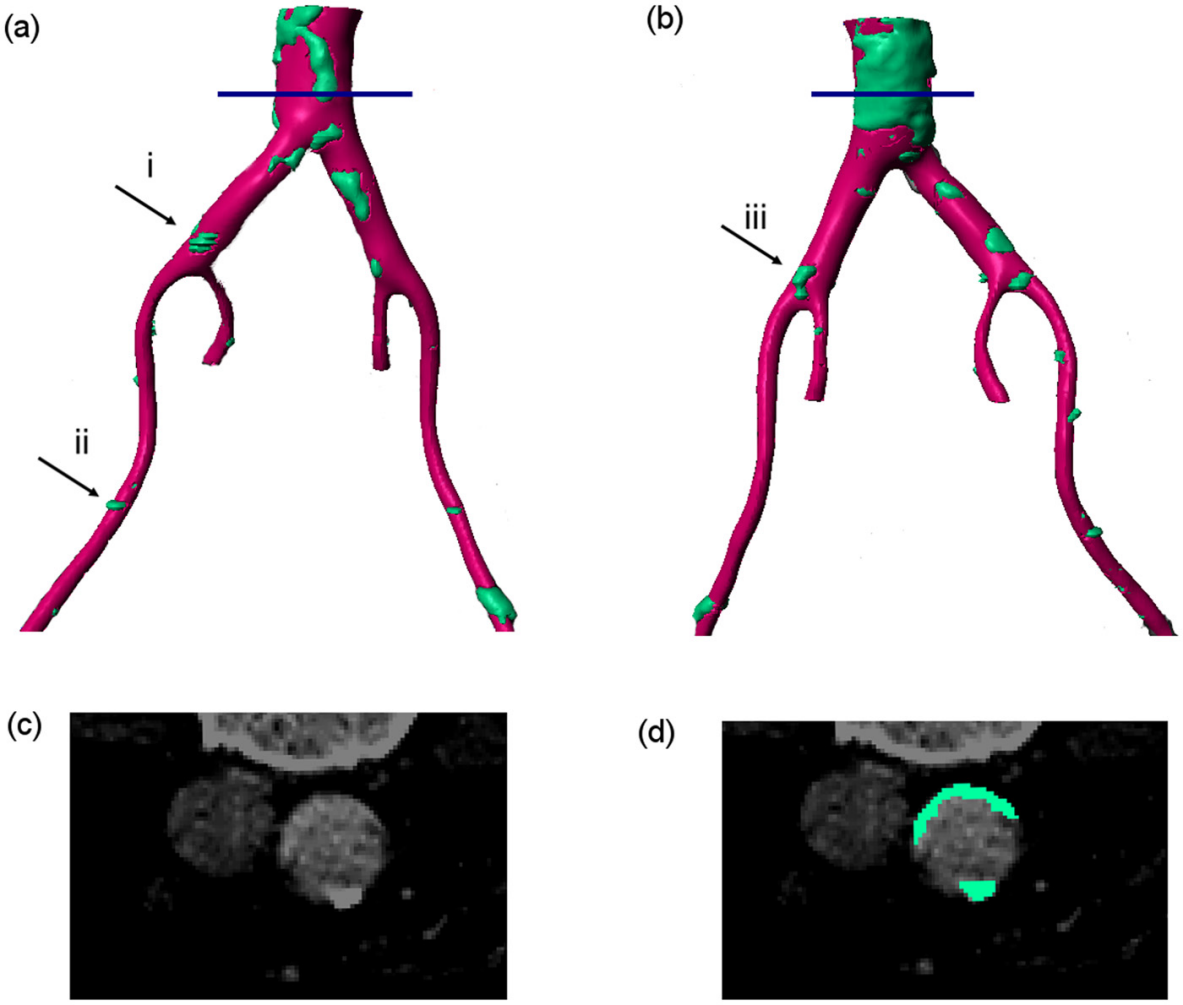
Reconstructed 3D geometry of the vessels around the iliac bifurcation. Pink regions show vessel wall while green regions show plaques/calcifications. (a) Left posterior view, (b) right anterior view, (c) shows the CT scan at the location indicated by the blue line in panels (a) and (b) and (d) shows the same slice with the plaque/calcification identified.

Due to the quality of the images, it was challenging to clearly distinguish the threshold between calcifications and plaques. To capture both calcification and plaque regions, intensity values of ⩾240 (same as bone) were selected to capture these solid locations, and a mask was assigned accordingly. The spine and bones were removed manually from the aforementioned mask, which resulted in the segmented geometry (in blue) shown in Figure 1.

**Figure 2.**
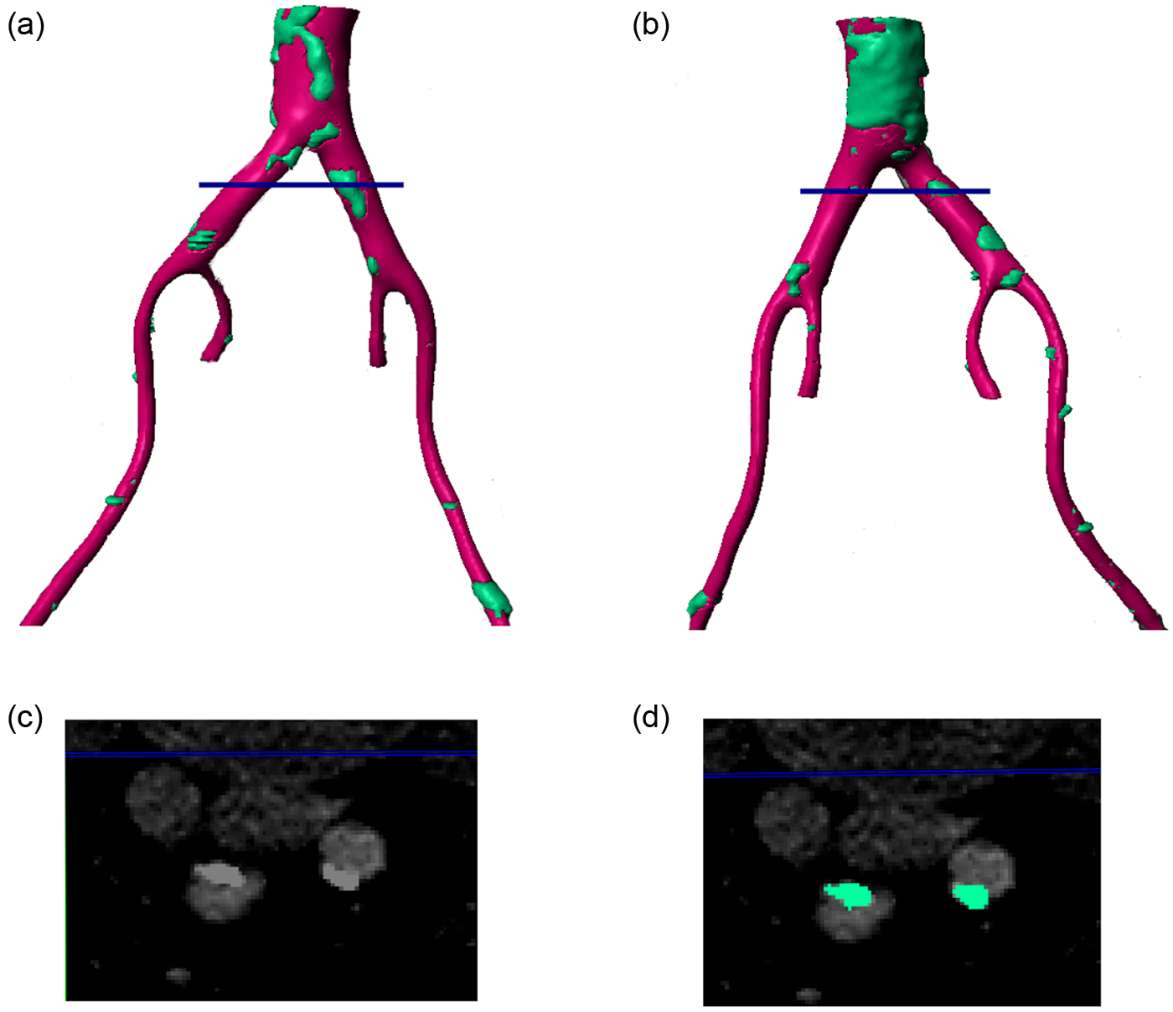
Reconstructed 3D geometry of the vessels around the iliac bifurcation. Pink regions show vessel wall while green regions show plaques/calcifications. (a) Left posterior view, (b) right anterior view, (c) shows the CT scan at the location indicated by the blue line in panels (a) and (b) and (d) shows the same slice with the plaque/calcification identified.

To capture the vessel wall, a lower intensity threshold was applied (⩾230), which included both the vessel wall (soft tissue) and the hard tissues (spine, bone and calcified regions). To only capture the vessel wall, the hard tissue mask that was previously generated (prior to the manual removal of the spine and bones) was deducted from the mask with the threshold of ⩾230 to only capture the soft tissue. An island removal algorithm and three-dimensional (3D) cropping were applied to remove the unrelated soft tissues such as kidneys and other isolated regions. Subsequently, a Gaussian smoothing filter with *σ* = 0.2 mm was applied in all three directions (*X, Y* and *Z*). The final mask can be seen as pink in Figures 1 and 2, with calcified regions indicated in blue.

Figure 1 shows both the soft and hard tissue mask for the extracted geometry. Figure 1(c) shows the original CT image containing both segmented and unsegmented regions at a plane indicated by the red line in Figure 1(a) and (b). As explained before and alluded to in the introduction, there is no definite threshold between plaque and calcification and thus the segmented area in Figure 1. After consultation and identification of relevant areas by the managing clinician and in an effort to illustrate the difference between calcification and plaque in descriptive terms, plaque regions are seen in the images as lighter with a deformed, closed, semi-elliptical shape while calcification regions show as slightly darker, thinner regions, very close to the wall of the lumen. Considering the vessel cross-section in Figure 1(c), it can be seen that this description indicates a plaque on the vessel wall at the lower edge (as the image is orientated) and a calcified region on the upper wall. Figure 1(d) shows these regions as identified by the mask.

Figure 2 provides a similar image, but with the cross-sectional plane defined downstream of the aortic bifurcation. In this case, each vessel shows a closed light shape, indicative of a plaque.

### Computational fluid dynamics

**Figure 3.**
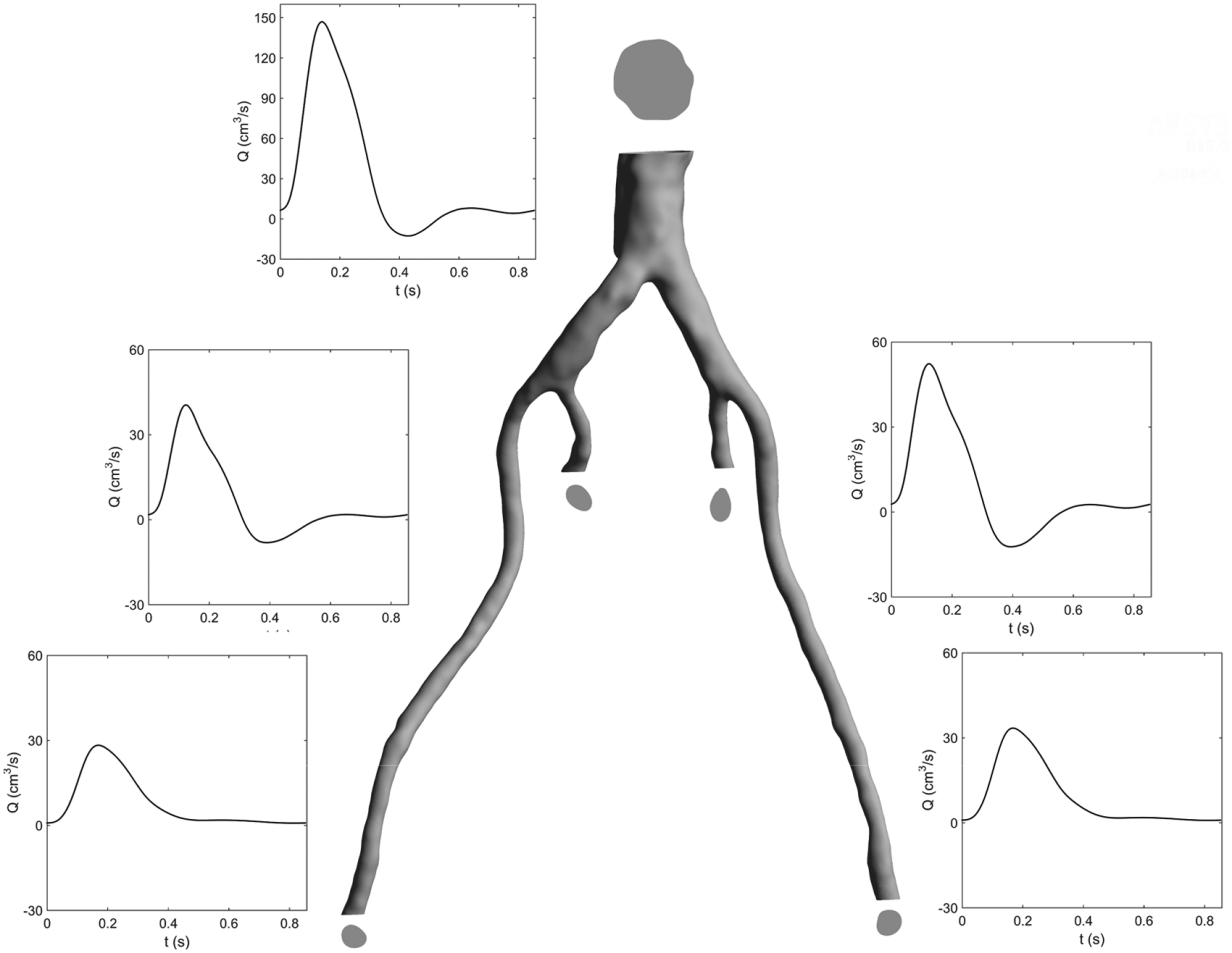
3D geometry used in CFD simulation, with boundary geometries and flow rates. Cross-sectional areas: distal abdominal aorta – 3.82 cm^2^, left internal iliac – 0.38 cm^2^, left external iliac – 0.34 cm^2^, right internal iliac – 0.41 cm^2^ and right external iliac – 0.39 cm^2^.

The 3D-segmented lumen was imported into ANSYS CFX (ANSYS Inc., Canonsburg, PA, USA), as shown in Figure 3. CFX uses the finite volume method to solve the Navier–Stokes equations numerically. The computational mesh was generated using ANSYS ICEM, and the mesh had ∼510,000 elements, of which approximately 31,000 were wall elements, with five prismatic layers used at the wall. Coarse (∼250,000 elements) and fine (∼1,000,000 elements) meshes were simulated in order to ensure that the results were relatively insensitive to the mesh size. The pressure and velocity fields change by a maximum of 3% between the coarse and medium meshes and by less than 1% upon further refinement. Hence, the medium mesh was deemed appropriate for this study.

The flow entering the segmented region (Figure 3) was extracted from a previous study,^17^ wherein invasive pressure data measured with a catheter was used to tune patient-specific boundary conditions (BCs). For the outlets, a constant pressure of 45 mmHg was applied to each boundary. This BC was selected as no clinical data were available for appropriately defining dynamic BCs, and flow split conditions force an unrealistic constant phase in the flow rate between branches.

Blood was considered to be an incompressible fluid with a density of 1056 kg/m^3^. To model the non-Newtonian properties of blood, the Carreau–Yasuda model was used with parameters defined by Gijsen et al.^18^ The flow was considered to be turbulent, using the hybrid *k–ε, k–ω*, shear stress transport turbulence model and a moderate inlet turbulence intensity of 1%. This model was chosen based on previous studies of blood flow in patients with dissected aortae, in which a small amount of turbulence was observed.^17^ Although the present vessels are downstream of the dissected region, it is likely that a low level of background turbulence remains in this region.

In the absence of detailed information about the vessel wall mechanical properties, the vessel wall was considered to be rigid. Further clarifications and a justification for this approach are presented in the ‘Discussion’ section. A time step of 5 ms was used, and the simulation was run for three cardiac cycles to achieve periodicity. Results from the third cycle were extracted and considered for further analysis.

### A multiscale, mechanistic model of endothelial mechanotransduction and LDL penetration

#### Transendothelial LDL transport

A mathematical model describing the processes related to the early stages of atherosclerosis was developed, which describes LDL transport from the artery lumen into the arterial wall, taking into account the effects of flow conditions on the endothelial cell layer and its pathways of volume and solute flux. The endothelium is modelled with a three-pore modelling approach taking into consideration the contributions of the vesicular pathway, normal junctions and leaky junctions. The fraction of leaky junctions is calculated as a function of flow conditions (e.g. WSS and OSI) and is used in conjunction with pore theory to determine the transport properties of this pathway. A recent analysis published by Kim and Giddens^19^ shows a similar model using time-average wall shear stress (TAWSS).

In the present model, the LDL transport equations are decomposed using three main penetration pathways: leaky junctions, normal junctions and vesicular pathways; so the bulk of volume flux (*J_v_*) through the endothelial membrane is given by

(1) $$J_{v}=J_{v,lj}+J_{v,nj}$$

where *J_v,lj_* is the flux through leaky junctions and *J_v,nj_* is the flux through normal junctions.

Assuming that a classical model of membrane transport can be used for transendothelial transport, the dissipation function Φ can be expressed as a function of the volume flux and the chemical potential^20,21^

(2) $$\Phi =\sum_{i=1}^{n}{\bar{J}}_{i}\cdot grad\left(-\mu_{i}\right)$$

where ${\bar{J}}_{i}$ is the volume flux and *µ_i_* is the chemical potential of each component in the solution. Under stationary conditions, assuming that ${\bar{J}}_{i}$ is constant at every point (*x*) in the membrane, the dissipation function for the membrane of thickness Δ*x* (in this case the endothelium) can be expressed as

(3) $$\Phi_{\Delta x}=\int_{0}^{\Delta x}\Phi dx$$

(4) $$\Phi_{\Delta x}=\int_{0}^{\Delta x}\sum_{i=1}^{n}{\bar{J}}_{i}\cdot \mathrm{grad}\left(-\mu_{i}\right)\mathrm{dx}$$

(5) $$\Phi_{\Delta x}=\sum_{i=1}^{n}J_{i}\cdot \Delta \mu_{i}$$

(6) $$\Phi_{\Delta x}=J_{w}\Delta \mu_{w}+J_{s}\Delta \mu_{s}$$

where the subscripts *w* and *s* denote the solvent and solute, respectively. Then, assuming that the chemical potentials at the membrane surfaces are identical to the solution chemical potential, Φ can be written as

(7) $$\Phi =\left(J_{w}{\bar{V}}_{w}+J_{s}{\bar{V}}_{s}\right)\Delta p+\left(\frac{J_{s}}{{\bar{c}}_{s}}\right)\Delta \Pi$$

where *J_w_* and *J_s_* are the fluxes of solvent and solute; ${\bar{V}}_{w}$ and ${\bar{V}}_{w}$ are the partial molar volumes of the solvent and the solute, respectively; Δ*p* is the pressure difference through the membrane, ΔΠ is the osmotic pressure and ${\bar{c}}_{s}$ is the mean concentration of solute. The dissipation function can be written then as a function of the volumetric flux (*J_v_*) and the diffusion flux (*J_D_*)

(8) $$\Phi =J_{v}\Delta p+J_{D}\Delta \Pi$$

After additional algebraic manipulations,^20^ from the previous equations, is it possible to obtain the classical Kedem–Katchalsky (KK) equations for *J_v_* and *J_s_*. So, in the specific case of the endothelium, the volumetric flux (*J_v_*) can be written as

(9) $$J_{v}=L_{p}\left(\Delta p_{\mathrm{end}}-\sigma_{d}\Delta \Pi \right)$$

Then, the volumetric flux through leaky junctions (*J_v,lj_*) is calculated using a modified version of the KK equations for membrane transport

(10) $$J_{v,\mathrm{lj}}=L_{p,\mathrm{lj}}\left(\Delta p_{\mathrm{end}}-\sigma_{d}\Delta \Pi \right)$$

(11) $$J_{s}=P_{i}\left(c_{\mathrm{lum}}-c_{w,\mathrm{end}}\right)\frac{\mathrm{Pe}}{e^{\mathrm{Pe}}-1}+J_{v}\left(1-\sigma \right)\bar{c}$$

(12) $$\mathrm{Pe}=\frac{J_{v}\left(1-\sigma \right)}{P_{i}}$$

where *L_p,lj_* is the hydraulic conductivity, Δ*p_end_* is the pressure difference through the endothelium, *σ_d_* is the osmotic reflection coefficient and ΔΠ is the osmotic pressure. The value of Δ*p_end_* is estimated by subtracting the externally applied pressure from either the average pressure throughout the domain (uniform pressure gradient) or the spatially varying time-average pressure at each wall location (time-averaged pressure gradient).

According to the *three pores* theory, solute flux (LDL flux in this case) only occurs through endothelial leaky cell (LC) junctions and vesicles

(13) $$J_{s}=J_{s,\mathrm{lj}}+J_{s,v}$$

assuming that the solute flux through the vesicular pathway (*J_s,v_*) is 10% of the solute flux through the leaky junction pathway (*J_s,lj_*).

#### Including the effect of endothelial cell shape

Endothelial cell shape will affect the amount of leaky junctions. As shown in previous work,^22,23^ the present model considers that under altered haemodynamics, endothelial cells do not have a typical cobblestone shape, but rather exhibit a more circular shape as well as increased permeability. The aim of the present approach is to propose a relationship between flow conditions (e.g. WSS and OSI) and endothelial permeability through the calculation of an Endothelial Cell Shape Index (ECSI). ECSI is related to the shape of the cells and takes values from zero to one, that is, a circle has an ECSI of one while a straight line has an ECSI of zero.

Some approaches based on ECSI calculated this variable as a function of WSS (for instance Olgac et al.^24^ used WSS values in steady state simulations). However, as previously mentioned, recent work shows that ECSI can be affected by other indices *as well*, such as OSI. Based on the seminal paper by Levesque et al.,^25^ a more recent work from Sáez et al.^26^ presented how a combination of OSI and TAWSS modifies the endothelial cell shape. This relationship will be the key for the modelling work presented here.

In this study, we will consider the role of two different Shear Stress Indices (SSI). These will be defined and described in detail in the next section.

Continuing with the cell-modelling aspects of this work, ECSI is defined according to

(14) $$\mathrm{ECSI}=a_{1}e^{a_{2}\mathrm{SSI}}+a_{3}e^{a_{4}\mathrm{SSI}}$$

LCs have high permeability to LDL, which can be linked to the magnitude of the SSI. Areas with high ECSI will be related to a higher number of mitotic cells (MCs), which are calculated as follows

(15) $$\mathrm{MC}=b_{1}e^{b_{2}\mathrm{ECSI}}$$

Assuming that, within the endothelium, the quantity of leaky MCs is approximately 80.5%, representing approximately 45.3% of the total number of LCs in that area,^27^ the number of LC is calculated as

(16) $$\mathrm{LC}=c_{1}+c_{2}\mathrm{MC}$$

Parameters *a*_1_, *a*_2_, *a*_3_, *a*_4_, *b*_1_, *b*_2_, *c*_1_ and *c*_2_ have been previously proposed for these equations^23^ (see Table 1 for numerical values). These parameters are based on various literature studies and are not patient-specific. However, it would not be possible to use patient-specific parameters in practical use of this model for predicting plaque locations, so the use of averaged literature values is necessary. Then, the ratio of endothelium (*ϕ*) covered by LCs is calculated as

(17) $$\phi =\frac{\mathrm{LC}\;\pi R_{\mathrm{cell}}^{2}}{\mathrm{unit}\;\mathrm{area}}$$

where *R_cell_* is the radius of a single cell. Finally, the total hydraulic conductivity of the endothelial leaky junctions (*L_p,lj_*) is defined as

(18) $$L_{p,\mathrm{lj}}=\phi \cdot L_{p,\mathrm{slj}}$$

where *L_p,slj_* is the hydraulic conductivity of a single leaky junction calculated as follows

(19) $$L_{p,\mathrm{slj}}=\frac{w^{2}}{3\mu l_{\mathrm{lj}}}$$

with *w* and *l_lj_* being the half-width and the length of the leaky junctions (see Table 1 for numerical values) and *µ* being the viscosity term used for the estimation of the LDL penetration.

**Table 1.** Parameters for the Transendothelial Penetration Model.

Parameter	Value	Units
*a*_1_	0.380	Dimensionless
*a*_2_	−0.79	Dimensionless
*a*_3_	0.225	Dimensionless
*a*_4_	−0.043	Dimensionless
*b*_1_	0.003739	Cells
*b*_2_	14.75	Dimensionless
*c*_1_	0.307	Cells
*c*_2_	0.805	Dimensionless
*R_cell_*	10	µm
*w*	20	nm
*l_lj_*	2	µm

#### Shear Stress Indices: SSI

In the permeability calculations described in the previous section, one of the inputs is an index indicative of the shear stress condition that leads to increased permeability (SSI). The TAWSS and OSI are two commonly used indices that are considered important for plaque formation. TAWSS describes the average magnitude of the shear stress, and the OSI gives an indication of the directionality of the shear stress, yielding 0 for uniaxial flows and 0.5 when there is no preferential direction. Typically, in permeability models for atherogenesis, TAWSS is used as the shear index related to permeability.

A number of studies^28–31^ have shown that regions with low average shear stress *combined* with high oscillatory shear stress have increased endothelial permeability along with other pathological responses. As previously mentioned, Sáez et al.^26^ presented the 3D remodelling of endothelial cells as the combined effect of OSI and TAWSS in a computational framework, which fitted experimental works presented before in in vitro studies.

In light of the indications for increased permeability in low, oscillatory regions, an index based on our own previous work is proposed,^32^ HOLMES (Highly Oscillatory, Low MagnitudE Shear), and it is given by

(20) HOLMES=TAWSS(0.5−OSI)

This parameter is equivalent to half the reciprocal of relative residence time (RRT), which was previously identified as a potential index for combining these two characteristics.^33^ The HOLMES indicator can be effectively understood as a modified TAWSS, with the (0.5−OSI) term further reducing the index in regions where the WSS is both low in magnitude and oscillatory in nature. Additionally, HOLMES provides a conceptually alternative explanation, offering a linear (rather than reciprocal) index proportional to TAWSS that intuitively corresponds to the observed effects of shear characteristics on endothelial permeability.

#### Estimating plaque location and metrics evaluation

As mentioned earlier and proposed previously, the magnitude of LDL fluxes across the wall along the artery can be used to identify atherosclerotic-prone regions. Given the close relationship of the volume flux (*J_v,lj_*) with the solute flux (*J_s_*) (see equation (11)), *J_v,lj_* is a sensible metric to identify plaque/calcification locations. Therefore, as in previous work,^32^ the volume flux, *J_v,lj_* will be used in this study. For simplicity, this will be noted *J_v_* herein.

Comparison of the calcification/plaque regions observed in the CT scans and those calculated by the chosen metric following the methodology shown was used to evaluate the efficacy of the model.

## Results

### TAWSS

WSS indices have shown promise in the study of atherosclerosis and plaque formation, as WSS greatly impacts the structure of the inner wall layer of the vessel. A number of WSS indices are commonly used in the analysis of blood flow simulations in order to describe the WSS characteristics as a single spatial distribution such as the TAWSS and the OSI. TAWSS is defined as

(21) $$\mathrm{TAWSS}=\frac{1}{T}\int_{0}^{T}\left|\tau \left(t\right)\right|\mathrm{dt}$$

where |τ(t)| is the magnitude of WSS vector at time *t*.^34^ The OSI is calculated as^34,35^

(22) $$\mathrm{OSI}=0.5\left(1-\frac{\left|\frac{1}{T}\int_{0}^{T}\tau \left(t\right)\mathrm{dt}\right|}{\mathrm{TAWSS}}\right)$$

**Figure 4.**
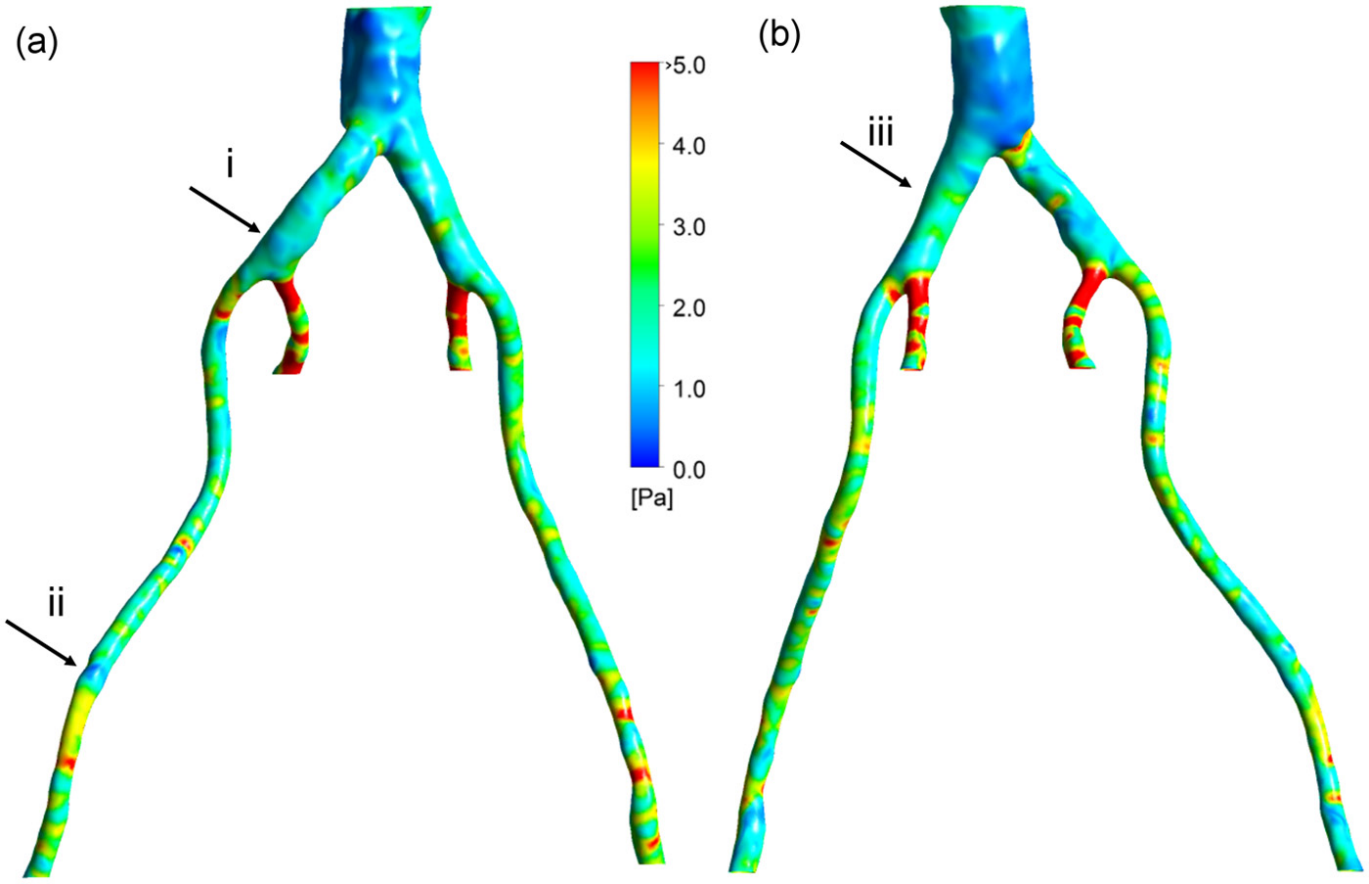
TAWSS: (a) left posterior view and (b) right anterior view.

The TAWSS distribution is shown in Figure 4 for both the posterior and anterior views. The TAWSS is at a maximum along the proximal internal iliac arteries, with values greater than 5 Pa. This is due to the fact that the diameters of these smaller vessels are reduced, which results in increased flow velocity and consequently higher TAWSS. The larger diameter in the external iliacs likewise results in lower TAWSS. In the distal abdominal upstream of the bifurcation, the TAWSS has low values.

### OSI

**Figure 5.**
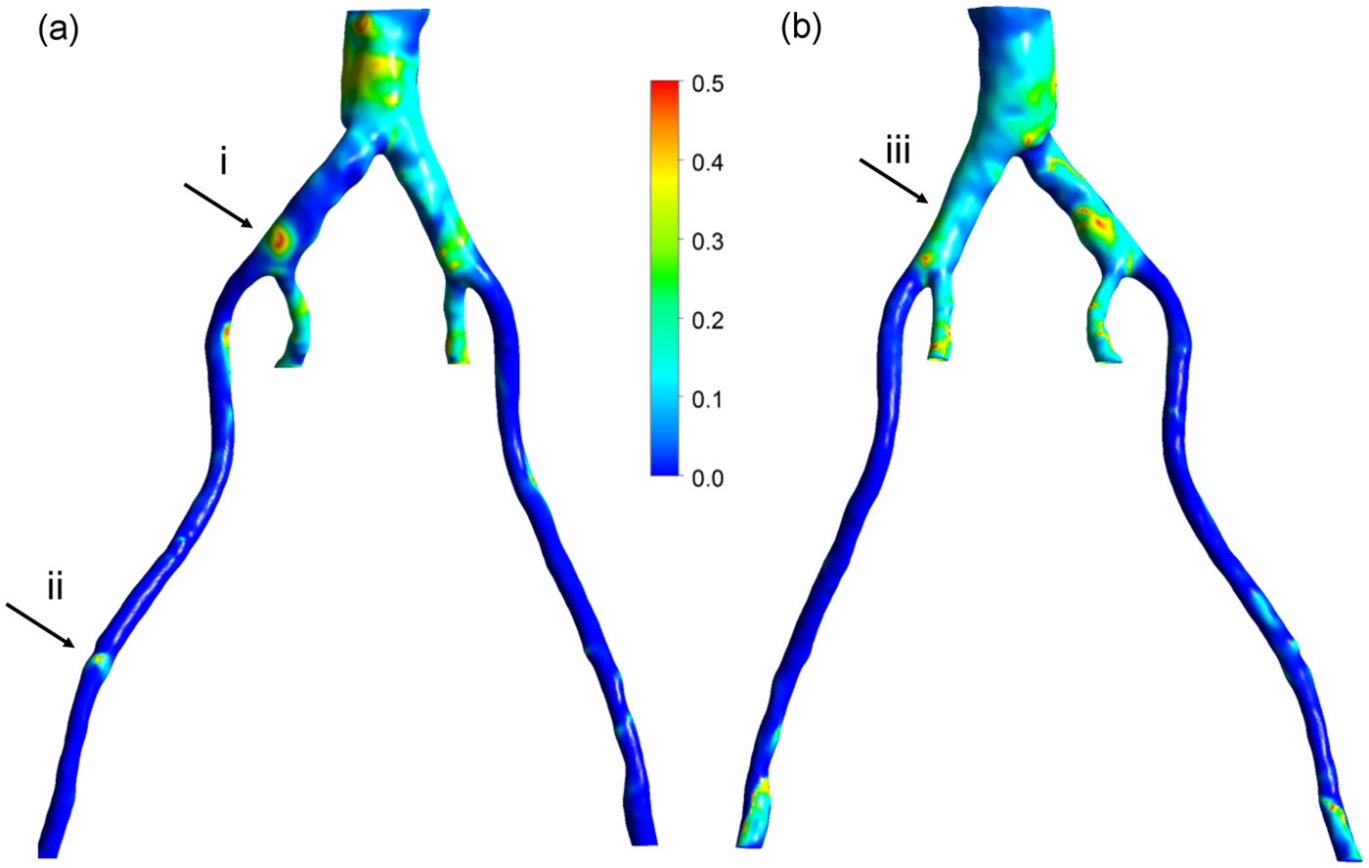
OSI: (a) left posterior view and (b) right anterior view.

The distribution of OSI is shown in Figure 5. The scattered, elevated regions of high OSI can be seen around the aortic bifurcation region, with values ⩾0.4. The OSI is higher upstream of the aortic bifurcation as a result of larger diameter, which shows that in some regions the blood flow does not have a preferential direction. Higher OSI values correspond to the locations where there are complex vortical structures. On the contrary, downstream of the aortic bifurcation, wherein the narrowing of the vessels occurs, lower values of OSI can be observed. This implies a more uniform blood flow stream corresponding to more structured streamlines in these regions. The inner iliac arteries still show high values of OSI, perhaps due to the nature of the vessel curvature in those regions.

### HOLMES

**Figure 6.**
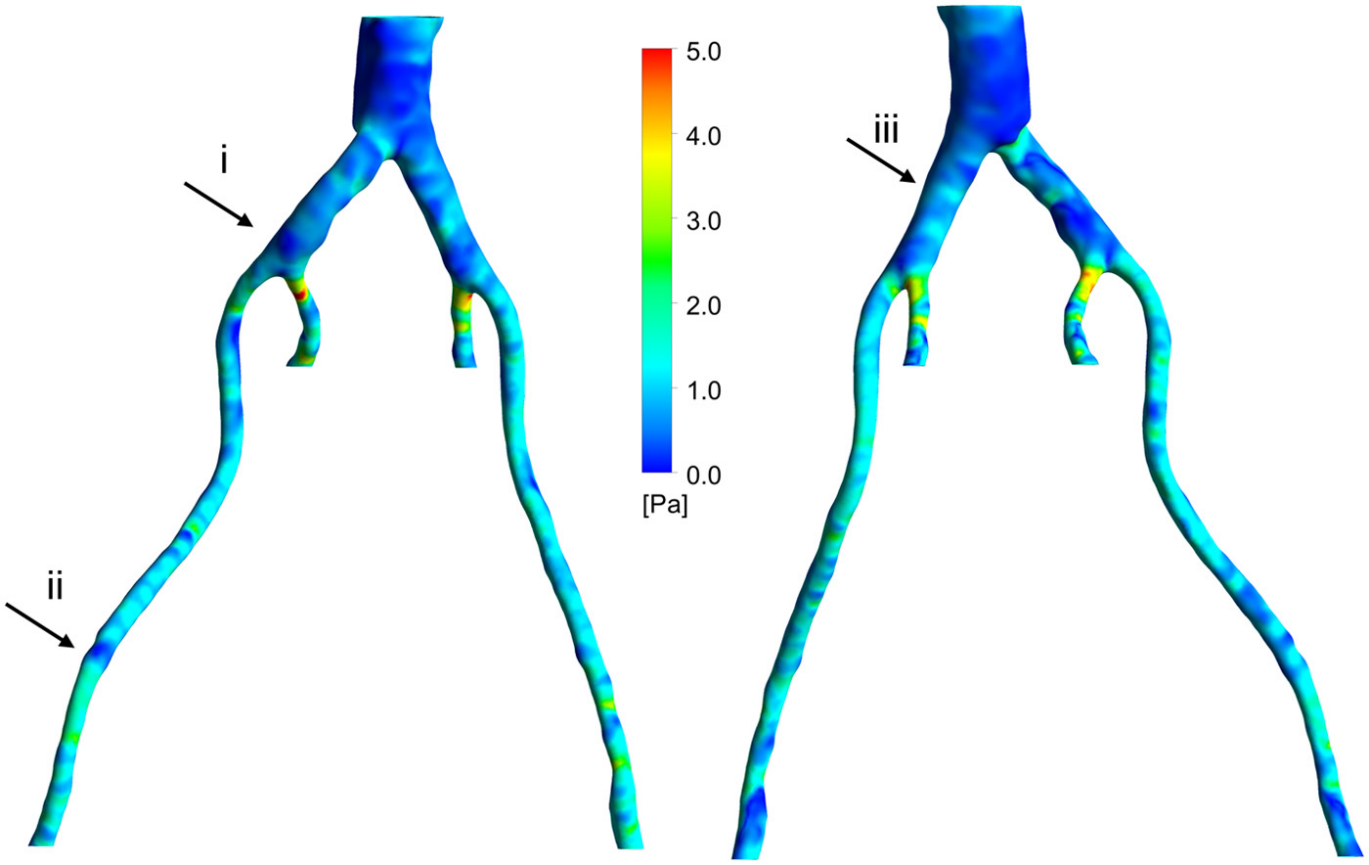
HOLMES: (a) left posterior view and (b) right anterior view.

Figure 6 shows the distribution of the HOLMES index. Note that regions of low HOLMES indicate regions at potential risk of damage. The lowest values are observed in a large region in the distal abdominal aorta and in regions in the iliac arteries. In comparison to the TAWSS, the HOLMES in the internal iliac arteries is fairly low, due to the high OSI in this region. The HOLMES in the external iliac arteries is similar to the TAWSS (although half the magnitude), except in the few regions where the OSI is significantly different from zero (e.g. arrow (ii)).

### Volume flux, *J_v_*

**Figure 7.**
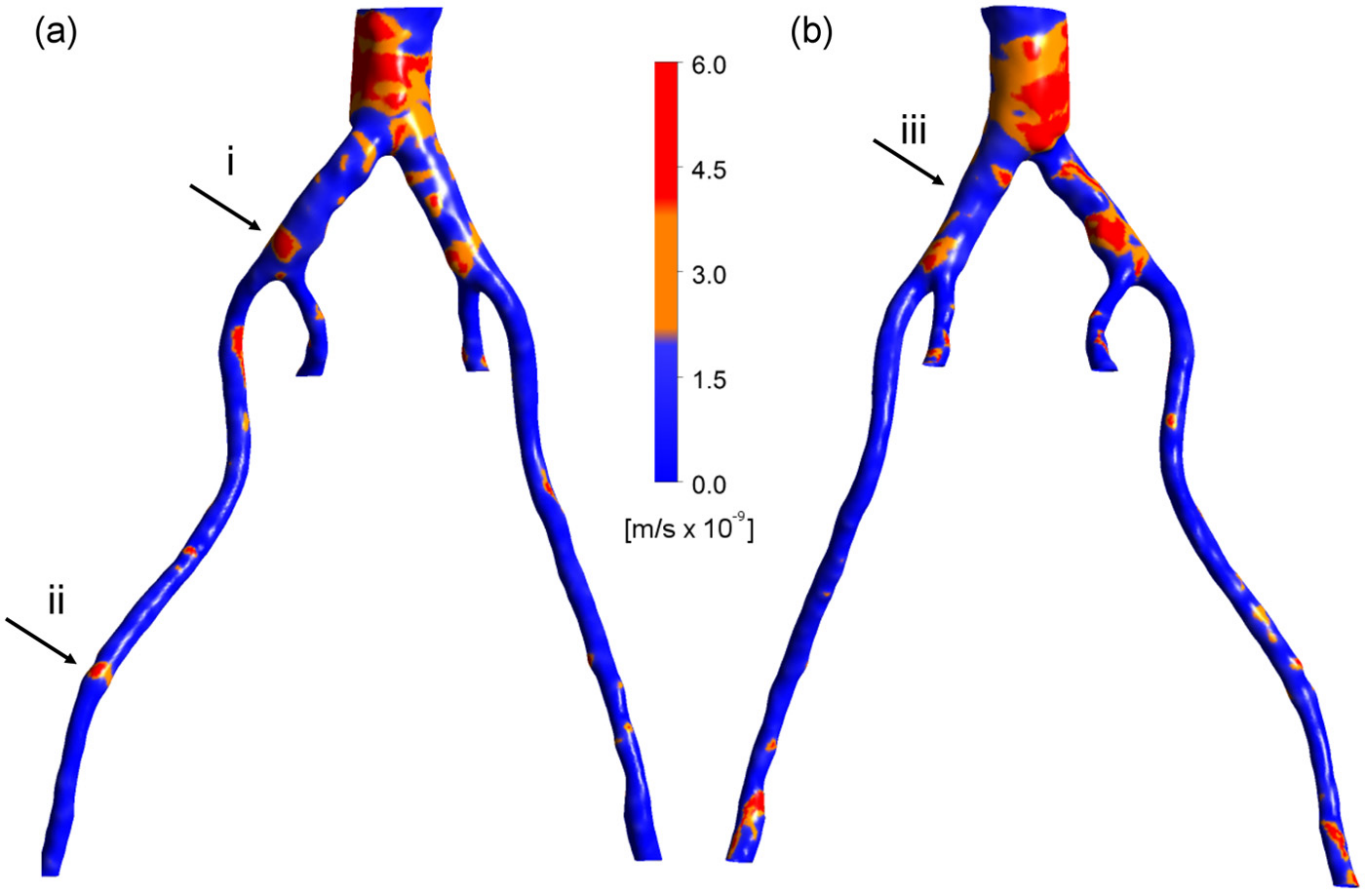
*J_v_*: (a) left posterior view and (b) right anterior view.

In light of the indications for increased permeability in low, oscillatory regions, the HOLMES index (see equation (20)) was used. Figure 7 shows the volume flux estimated from the calculations of ECSI and the hydraulic conductivity of leaky junctions at each mesh location on the arterial wall. As explained previously, there is no exact threshold to distinguish the plaque from calcification, with the exception of the nature of the plaque’s geometry (closed shape). Regions where values of *J_v_* ⩾ 2 × 10^−9^ m/s are considered to represent both plaques and calcifications, as shown in orange and red in Figure 7, respectively. However, the guidelines described above can be used to identify plaques from calcifications. It transpires that regions where *J_v_* exceeds ∼4 × 10^−9^ m/s appear to correspond to plaques visible in Figures 1 and 2. Although the exact shapes of these regions are not predicted exactly, elevated regions of *J_v_* correspond well with the *location* of plaques and calcifications. Please note that this is a model that focuses on the prediction of lesion sites, not plaque growth (which would include an additional component of remodelling of the arterial wall on top of identifying correct plaque/calcification regions).

To highlight the efficacy of the plaque locations, regions of plaques as predicted from *J_v_* > 4 × 10^−9^ m/s can be identified in the right anterior view in the distal aorta, in the left common iliac artery, in the right common iliac artery just before the secondary bifurcation and in the distal right external iliac. Comparing Figure 7 with Figures 1 and 2, it can be seen that in these regions, the vessel wall gave a similar CT signal to regions of hard tissue. More scattered, smaller regions of hard tissue can be identified in Figures 1 and 2, which are captured by *J_v_* in some cases and not in others. Similarly, considering the left posterior view, there are possible plaques in the distal aorta (high *J_v_*), just upstream of the secondary bifurcation in the left common iliac artery, just proximal to the aortic bifurcation and in the distal left external iliac, which compare well with hard regions in Figures 1 and 2. However, a large hard tissue region in right external iliac does not correspond to elevated *J_v_*. Nonetheless, the majority of the regions of plaques and/or calcifications observed in the CT scans are predicted by regions of elevated *J_v_*.

Please note that the regions in Figures 1 and 4–7 identified as (i), (ii) and (iii) will be used in the ‘Discussion’ section.

**Figure 8.**
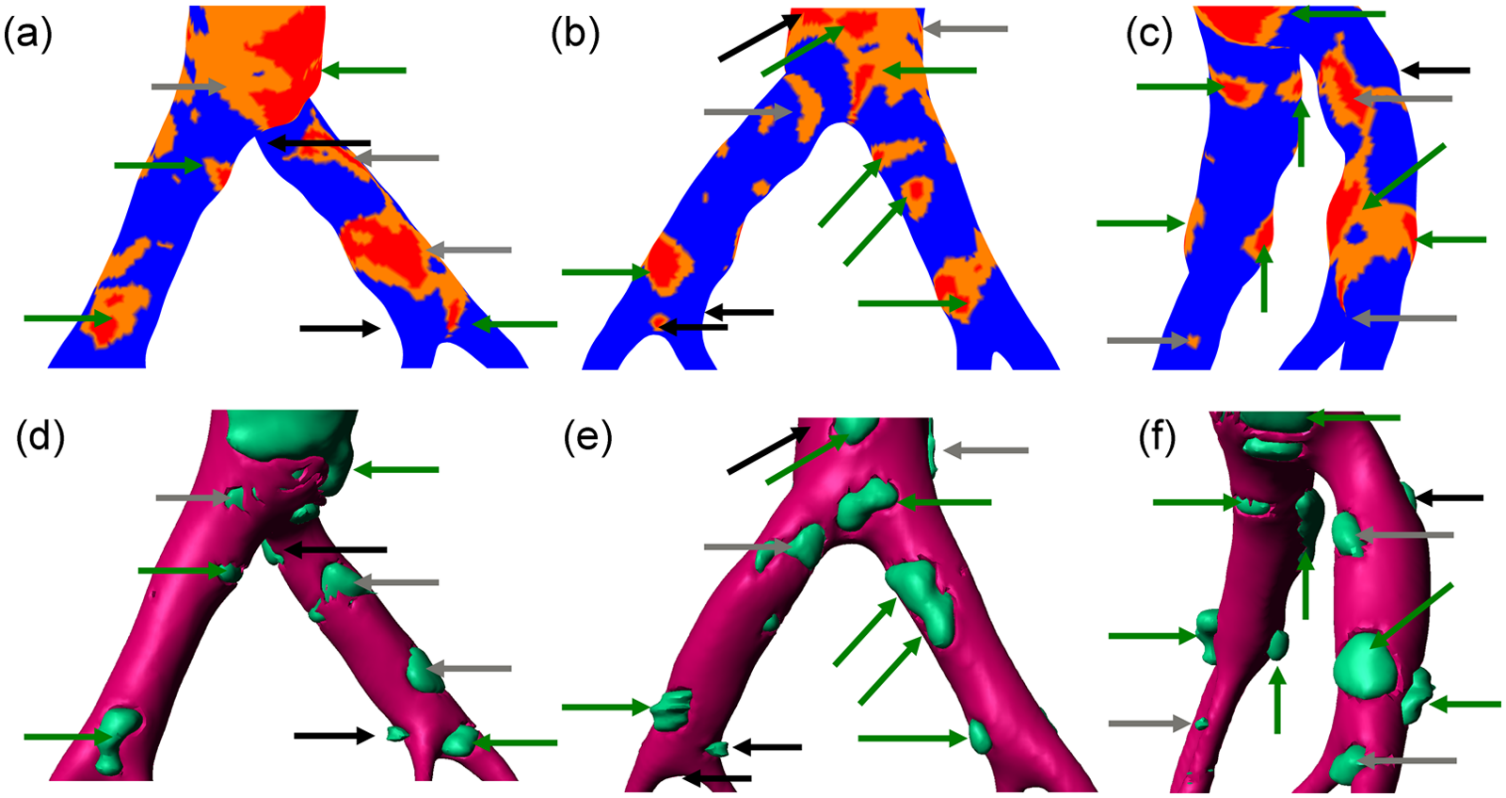
Comparison between predicted (top row) and observed (bottom row) plaque locations around the iliac bifurcation. Arrows indicate plaques, with colour coding to rate the quality of the match between the simulated *J_v_* and the clinical data: green – good match, grey – reasonable match and black – failed to match. (a) and (d) Right anterior view, (b) and (e) left posterior view and (c) and (f) view from right side.

Figure 8 provides a more detailed view of the results predicted by the model, through investigation of the aortic bifurcation region from several angles. Arrows are used to identify plaques and coloured to provide a qualitative evaluation of the efficacy of the model in accurately *locating* plaques. Note that by arbitrarily defining a single threshold for a plaque/calcified region in both the clinical and the simulation data, the thresholding approach precludes accurate identification of the shape of plaques, but enables only identification of the location.

Comparing first Figure 8(a) and (d), in the right anterior view, nine independent plaque regions can be observed in the clinical image. Of these, four are very well match in term of location by the simulation, as indicated by the corresponding green arrows in Figure 8(a). A further three plaques are identified reasonably well. For example, the small plaque in the top left-hand corner is within a region of elevated *J_v_*, and the two grey arrows on the right common iliac artery are close to similar regions of elevated *J_v_*. The two black arrows on the right common iliac artery point to plaques apparent in the clinical image without a corresponding region of elevated *J_v_* predicted by the simulation. These are what we can call ‘false negatives’. In Figure 8(b) and (e), showing the left posterior view, a further 11 arrows indicate plaques, of which the 6 green arrows are a good match, the 2 grey arrows show a reasonable match and the 3 black arrows shows failed matches. In this case, there is one false negative (on the inner wall of the right common iliac artery) and two false positives (in the distal aorta and at the secondary bifurcation in the right common iliac artery). In the view from the right side (Figure 8(c) and (f)), 11 arrows can be seen. From this view, seven are good matches, three are ok matches and one false negative is observed. While this analysis is not exhaustive, it demonstrates the efficacy of the model in predicting plaque locations. As an indication of the quality of the fitting, of the total 31 arrows, 55% are green, 26% are grey and 20% are black, of which 13% are false negatives and the remaining 7% are false positives. Overall, albeit rather qualitatively, the technique appears to be effective over 75% of the time. This will be further elaborated upon in ‘Discussion’ section.

## Discussion

As mentioned in the introduction, much work has been done on plaque location focused on haemodynamic indices alone. The results of this study demonstrate that there is a way forward for clear, interpretable and biology-based metrics of plaque location through multiscale, multiphysics models, combining both biomechanical and biochemical aspects of calcification and plaque formation. While a large-scale study with multiple patients would be required in order to estimate the sensitivity and specificity of the model, the results of this study show that there a significant proportion of the identified plaque regions corresponding harder wall regions, and hence possibly plaques, in the clinical imaging data (Figure 8). There are a few regions of elevated *J_v_* that do not correspond to calcifications or plaques, and vice versa, but the relative proportion is small. This is no small feat and the results are very promising, considering the inexact classification of such regions, uncertainty from the CT images in the identification of plaque and the nature of the anatomical description and the limitations of the modelling approach.

The utility of a mechanistic, multiscale interpretation for plaque location using the HOLMES index rather than the more common TAWSS, in combination of a model of endothelial mechanotransduction leading to *J_v_*, can be observed by considering the regions identified by arrows in Figures 1 and 4–7. In these figures, arrows indicate three regions for consideration, (i) the right common iliac artery, (ii) the right external iliac artery and (iii) the left common iliac artery. At location (i) in Figure 1, a structure can be observed that may represent a plaque. The TAWSS at this location (Figure 4) is low, but not particularly so. However, the OSI (Figure 5) is very high in this region, thereby decreasing the HOLMES index (Figure 6) considerably, leading to a predicted high *J_v_* value (Figure 7). In location (ii), a localised region of low TAWSS and high OSI can be observed, yielding a low HOLMES and thus high *J_v_* was predicted, as observed in Figure 7. Figure 1 also shows a potential plaque at this location. Finally, at location (iii), no plaque or calcification is observed in Figure 1. The TAWSS in this region is not significantly different to location (i) (Figure 4), but the OSI is lower, compared to region (i) (Figure 5). Hence, the HOLMES is moderate and *J_v_* is low, as would be expected where calcification is absent. These three regions demonstrate the reasons for the efficacy of the use of a multiscale, multifactorial approach including a combined haemodynamic indicator (HOLMES), in that regions characterised by combined low magnitude and oscillatory shear stress lead to elevated *J_v_*.

The current simulations used a simplified fluid dynamics model, in which the vessel wall motion was not considered in the simulation and simple constant pressure BCs were applied. A detailed sensitivity mesh analysis was then performed for the fluid simulation, leading to choosing the mesh described in the methods as an optimal one. In preliminary simulations, a fluid–structure interaction (FSI) model was investigated for the present geometry, using vessel wall parameters from Raghavan and Vorp;^36^ however, the results showed no significant differences in the flow results and derived haemodynamic wall-related parameters in this case, and the simulation time was impractically long for finer meshes. The compliance of the vasculature in AD patients has been observed to be altered,^37^ and hence quantification of vessel wall parameters in healthy patients is not applicable to this study. Furthermore, the observed calcification would likely alter local wall properties.

Results may differ in other arterial locations of increased compliance (e.g. the main aorta), which is a flow case that has been studied by the authors in the context of AD.^38^ However, the difficulty of the set-up and the effects of parameter uncertainty were significantly increased and simulation time increased more than 10-fold. FSI simulations utilising detailed knowledge of the vessel wall properties might further increase the accuracy of the simulations. Likewise, if detailed BC data were available, then the accuracy of the fluid dynamics simulation would likely be improved. However, given the comparison with preliminary FSI results for this region, computational time and difficulty of set-up, the model used here was deemed as a very-good compromise as the parameters used in this study enabled relatively successful identification of the plaque/calcification regions, indicating that such modifications to the mechanical aspects of the simulation would likely only provide incremental improvements to the predictive capability of the model at the expense of a significant increase in complexity and computational expense.

Future work will involve longitudinal studies of a small cohort of patients to further analyse the predictive ability of the model and to establish how dependent the model efficacy is on model parameters; this will include additional methods for plaque quantification for the comparison of simulation results with in vivo data. Recent work^39,40^ shows the development of new statistical methods to establish quantitative correlations between simulations and in vivo data with respect to plaque location. These are approaches that we would be keen to use for future, multipatient studies. We would like to emphasise, however, that the focus of this article is on the use and potential of multiscale modelling and simulation tools and the development of interpretable, physiologically-based metrics to understand plaque location.

## Conclusion

In this study, regions of vessel wall calcification and plaque formation were predicted using a multifactorial, multiscale modelling approach. The method combined fluid dynamics simulation with a mathematical model of transendothelial LDL transport, in order to estimate the volume flux through leaky junctions. The predicted distributions of the volume flux were compared with the regions of the vessel wall corresponding to calcified regions and plaques, as interpreted from the original CT scans. The interface between the fluid dynamics and biochemical models is the WSS index, which is used to estimate an ECSI, which influences the hydraulic conductivity and hence the volume flux through the endothelium (*J_v_*). Rather than the typical TAWSS or OSI, a combined index, termed HOLMES, was used to emphasise regions of highly oscillatory, low-magnitude WSS, in agreement with several studies implicating this WSS environment as particularly deleterious for the endothelium. The results showed that the model was able to predict the majority of the sites of calcification observed in the CT scan images and only failed to identify a few small regions. These results highlight the benefits of using a multiscale, multifactorial approach to simulating cardiovascular diseases, rather than focusing only on the mechanics or the biology. Future large-scale studies will enable identification of stricter guidelines for identifying plaques/calcified regions, and thereby further increase the predictive power of the model.
